# Review of the selected proceedings of the Fifth International Workshop on Data Integration in the Life Sciences 2008

**DOI:** 10.1186/1471-2105-9-S8-S1

**Published:** 2008-07-22

**Authors:** Amos Bairoch, Sarah Cohen-Boulakia, Christine Froidevaux

**Affiliations:** 1 Swiss Institute of Bioinformatics (SIB) and Department of Structural Biology and Bioinformatics, University of Geneva, Switzerland; 2 Laboratoire de Recherche en Informatique (LRI), CNRS UMR 8623, Université Paris-Sud 11, France

## Background

For several years now, there has been an exponential growth of the amount of life science data (e.g., sequenced complete genomes, 3D structures, DNA chips, Mass spectroscopy data) most of which are generated by high throughput experiments. This exponential corpus of data is stored and made available through a large number of databases and resources over the web, but unfortunately still with a high degree of semantic heterogeneity and varying levels of quality. These data must be combined together and processed by bioinformatics tools deployed on powerful and efficient platforms to permit the uncovering of patterns, similarities and in general to help in the process of discovery. Analysing complex, voluminous, and heterogeneous data and guiding the analysis of data are thus of paramount importance and necessitate the involvement of data integration techniques.

## DILS 2008 venue

DILS 2008 was the fifth in an international workshop series that aims at fostering discussion, exchange, and innovation in research and development in the area of data integration for the life sciences. The DILS 2008 workshop was held at the University of Evry, in what is known as the 'Genomic Valley' at the heart of the Ile-de-France region, in France. Each previous DILS workshop attracted around 100 researchers from all over the world and has seen an increase of submitted papers over the preceding one. This year was not an exception and the number of submitted papers increased to 54. The 18 papers selected for presentation at DILS 2008 by the Program Committee cover a wide spectrum of theoretical and practical issues including data annotation, semantic web for the life sciences, and data mining on integrated biological data. 16 of them have been published in the Volume 5103 of *Lecture Note in BioInformatics of Springer-Verlag*, the two remaining papers have been chosen for publication in this supplement to *BMC Bioinformatics*.

## Summary of the selected contributions

The two papers selected for *BMC Bioinformatics* are extended and improved versions of the best papers accepted to DILS 2008. In the following paragraphs, we briefly review them.

The research paper by Jaeger *et al*. [1] addresses the challenging problem of functional annotation of proteins. The methods they designed and developed identify conserved protein interaction graphs and predict missing protein functions from orthologs. Their contribution is two-fold. On the one hand, the procedure they have developed has shown its ability to retrieve more than 80% of the GO annotations for UniProtKb/Swiss-Prot proteins with highly conserved orthologs. On the other hand, new GO annotations have been predicted on a subset of proteins. Results have been validated by biological experts.

The system paper by Jenkinson *et al*. [2] presents the latest updates of the Distributed Annotating System (DAS), increasingly used in the life science community. Extensions presented include dealing with various data types and providing an ontology for protein features. These new functionalities make the latest release of DAS able to span several areas, from genomic sequences to protein interactions.

## Workshop Program

In addition to the 18 presented papers, DILS 2008 featured three keynote talks by Olivier Bodenreider, National Library of Medicine, NIH, USA; Peter Karp, SRI International, USA; and Norman Paton, University of Manchester, UK. DILS 2008 also included a tutorial on Bio-ontologies and a session dedicated to updates of biomolecular resources of world-wide importance: the UniProt knowledgebase and the EBI proteomics services.

## Competing interests

The authors declare that they have no competing interests.

## Authors' contributions

All authors wrote and approved the manuscript.
